# Epidermal growth factor regulates the development of stem and progenitor Leydig cells in rats

**DOI:** 10.1111/jcmm.15302

**Published:** 2020-05-22

**Authors:** Xiaoheng Li, Yiyan Wang, Qiqi Zhu, Kaiming Yuan, Zhijian Su, Fei Ge, Ren‐Shan Ge, Yadong Huang

**Affiliations:** ^1^ Department of Cell Biology & Guangdong Provincial Key Laboratory of Bioengineering Medicine Jinan University Guangzhou China; ^2^ Department of Anesthesiology the Second Affiliated Hospital and Yuying Children's Hospital of Wenzhou Medical University Wenzhou China

**Keywords:** EGF, StAR, stem/progenitor Leydig cells, steroidogenesis, steroids

## Abstract

Epidermal growth factor (EGF) has many physiological roles. However, its effects on stem and progenitor Leydig cell development remain unclear. Rat stem and progenitor Leydig cells were cultured with different concentrations of EGF alone or in combination with EGF antagonist, erlotinib or cetuximab. EGF (1 and 10 ng/mL) stimulated the proliferation of stem Leydig cells on the surface of seminiferous tubules and isolated CD90^+^ stem Leydig cells and progenitor Leydig cells but it blocked their differentiation. EGF also exerted anti‐apoptotic effects of progenitor Leydig cells. Erlotinib and cetuximab are able to reverse EGF‐mediated action. Gene microarray and qPCR of EGF‐treated progenitor Leydig cells revealed that the down‐regulation of steroidogenesis‐related proteins (*Star* and *Hsd3b1*) and antioxidative genes. It was found that EGF acted as a proliferative agent via increasing phosphorylation of AKT1. In conclusion, EGF stimulates the proliferation of rat stem and progenitor Leydig cells but blocks their differentiation.

## 1 INTRODUCTION

The androgen deficiency could be treated by transplanting stem (SLC)/progenitor (PLC) Leydig cells (LCs) into the host testis.^1^ Androgen production not only depends upon LC number but also on its maturity.^2^ The development of LCs starts from SLCs.^1^ A set of testicular SLCs in the neonatal and adult rat,^3^, ^4^ mouse^5^, ^6^, ^7^ and human^8^ testes were identified. During puberty in rats, the SLC commits into a spindle‐shaped PLC around days 12‐14 postpartum.^9^, ^10^ PLCs begin to express some steroidogenic enzymes, including cytochrome P450 cholesterol side‐chain cleavage enzyme (CYP11A1), 3β‐hydroxysteroid dehydrogenase isoform 1 (HSD3B1) and cytochrome 17α‐hydroxylase/17,20‐lyase (CYP17A1).^9^ However, PLCs do not express the last‐step androgen synthetic enzyme, 17β‐hydroxysteroid dehydrogenase isoform 3 (HSD17B3).^9^ PLCs also express high levels of androgen metabolizing enzymes, including steroid 5α‐reductase 1 (SRD5A1) and 3α‐hydroxysteroid dehydrogenase.^9^ Androstenedione made by PLCs after CYP17A1 catalysis was metabolized into androstanedione by SRD5A1 via 5α‐reduction and further into androsterone (AO) via 3α‐reduction, thus being the primary androgen secreted by PLCs.^9^ PLCs undergo transitions into oval lipid‐droplet‐rich immature Leydig cells (ILCs) around days 28 postpartum, when ILCs appear to express HSD17B3 to synthesize testosterone (T).^9^ ILCs still have higher levels of androgen metabolizing enzymes, and they metabolize T into dihydrotestosterone via 5α‐reduction by SRD5A1 and further into 5α‐androstane‐3α,17‐diol (DIOL) via 3α‐reduction.^9^ After PLCs enter the ILC stage, an important glucocorticoid‐metabolizing 11β‐hydroxysteroid dehydrogenase 1 (HSD11B1) is expressed and is exclusively located in ILCs and adult Leydig cells (ALCs).^11^, ^12^ ILCs differentiate into ALCs around days 56 postpartum.^9^, ^13^ ALCs mainly secrete T because of the disappearance of SRD5A1 protein.^9^

Leydig cell development during puberty mainly relies on the regulation of luteinizing hormone (LH),^14^, ^15^ because PLCs, ILCs and ALCs all contain its receptor (LHCGR).^2^, ^16^ Indeed, LH can stimulate PLC proliferation and differentiation.^9^, ^14^, ^15^, ^17^ However, SLCs do not express LHCGR and do not need LH for the regulation.^3^, ^4^ SLC proliferation and differentiation depend on signalling pathways acting between somatic and stem cells in rodents and humans during the perinatal and prepubertal period.^18^, ^19^ Many growth factors may have an impact on the development of LCs during puberty.^1^ One of these growth factors could be epidermal growth factor (EGF). Evidence points to the regulation of EGF for the development of steroid‐producing gland. EGF has been found to promote functional maturation of the foetal primate adrenal glands.^20^ EGF plays a role in the development of testis because EGF knockout mice are sterile.^21^ Despite the physiological importance of EGF in testicular function, its mechanism of action on LC development remains unknown.

Epidermal growth factor is a growth factor that was first purified from mouse salivary glands.^22^ It exerts numerous roles in different cell types. EGF exerts its action through binding to its transmembrane receptors, EGF receptors, thus stimulating proliferation, growth and differentiation of many types of cells.^22^ Previous studies have demonstrated the presence of EGF receptors in human, bovine, rat and mouse LCs, suggesting that EGF has a role in LCs.^23^, ^24^, ^25^, ^26^

However, the effects of EGF on the development of SLCs/PLCs into the LC lineage are largely unclear. In our previous studies, we established a model for studying SLC development using an in vitro culture of LC‐depleted seminiferous tubules (STs).^4^, ^27^ ALCs not SLCs can be eliminated after intraperitoneal injection of 75 mg/kg ethane dimethane sulfonate (EDS) into rats.^28^, ^29^ LC‐depleted ST is isolated for the serum‐free culture. In this system, SLCs on the surface of STs can be induced to differentiate into the LC lineage under the LC differentiation medium (LDM) containing LH and insulin‐transferrin‐selenium supplement (ITS).^4^, ^27^, ^30^ In the current study, we examined the effects of EGF on the development of SLCs/PLCs.

## 2 MATERIALS AND METHODS

### 2.1 Chemicals

Bovine serum albumin (BSA), ITS, dimethyl sulfoxide (DMSO), EGF, Percoll, M199, DMEM, F12 and HBSS medium were obtained from Sigma‐Aldrich. Click‐it EdU Alexa Fluor was obtained from Life Technologies. Erlotinib HCl (E), an EGFR kinase inhibitor,^31^ was obtained from Selleck. LH was a gift of NIDDK (US). Cetuximab (Cet) was obtained from MCE (Cat No: HY‐P9905). EDS was purchased from Pterosaur Biotech. [^3^H]‐Thymidine, [^3^H]‐androsterone ([^3^H]‐AO) and [^3^H]‐testosterone ([^3^H]‐T) tracers were obtained from DuPont‐New England Nuclear. Hyamine hydroxide was purchased from ICN Radiochemicals. Primer information was listed in Table S1. Antibody information was listed in Table S2.

### 2.2 Animals

Male Sprague‐Dawley rats were purchased from Shanghai Laboratory Animal Co. Ltd. Forty male rats at age of 7 days were used for the isolation of CD90^+^ SLCs each time. Forty male rats at age of 21 days were used for the isolation of PLCs each time. Male rats at age of 90 days were used for ST isolation and culture of SLCs. The animal procedure was approved by the Institutional Animal Care and Use Committee of Wenzhou Medical University and was performed in accordance with the Guide for the Care and Use of Laboratory Animals.

### 2.3 Purification and culture of CD90^+^ SLCs

Testicular CD90^+^ cells were thought to be the SLCs.^30^ Purification and culture of CD90^+^ SLCs from 7‐day‐old male rats were performed. Peritubular testicular cells were obtained from collagenase (0.1 mg/mL)‐treated isolated STs and stained using CD90 antibody, and purified using BD IMag™ bead. Cells were incubated with CD90 antibody (1:100) in BD IMag™ Buffer for 20 minutes on ice. Beads were incubated with CD90‐conjugated cells for 30 minutes. After washing, the cells were separated by BD IMagnet™ for 10 minutes. Cells were suspended in M199 medium. The purity of CD90^+^ cells (SLCs) was over 99%. To study the proliferation of SLCs, CD90^+^ cells (1 × 10^4^ cells/well) in LDM medium were seeded in 12‐well plate and incubated with control (LDM), EGF (10 ng/mL), Cet (an EGF antagonist, 5 µg/mL) and EGF (10 ng/mL)+Cet (5 µg/mL) for 24 hours. Then, cells were washed using PBS, and EdU incubation was performed as following section. Our previous study has demonstrated that the SLCs are cultured during the first week of culture, and the number of SLCs is greatly amplified.^30^ Then, SLCs were switched to LDM for additional 14 days, the amplified SLCs could be differentiated into ALCs, and thus, the increased number of ALCs could contribute into the robust increase of T level in the medium.^30^ Using this approach, EGF (0, 10 ng/mL) with or without EGF antagonist (Cet, 5 μg/mL) was added to SLCs (1 × 10^4^ cells/well) in M199 and cultured at 34°C and 5% CO_2_ for 7 days, and then, SLCs were switched into LDM for additional 14 days. Media were collected for the measurement of T level.

To study the differentiation of SLCs, CD90^+^ cells (1 × 10^4^ cells/well) in LDM medium were seeded in 12‐well plate and incubated with control (LDM), EGF (10 ng/mL), Cet (an EGF antagonist, 5 µg/mL) and EGF (10 ng/mL)+Cet (5 µg/mL) for 14 days. Media were collected for the measurement of T level.

### 2.4 ST isolation and culture of SLCs on the surface of STs

The procedure for ST isolation and culture was performed as previously described.^27^, ^30^ Briefly, EDS was dissolved in a 1:3 solution mixture of DMSO and water. One 90‐day‐old rat was selected and injected intraperitoneally with a single dose (75 mg/kg body weight) of EDS, which can effectively eliminate all LCs in the testis without damaging SLCs.^12^, ^13^ Seven days after EDS, LCs were all eliminated.^32^ The rat was euthanized under CO_2_. Two testes were taken out and placed in cold M199 medium and decapsulated. STs were mechanically separated using a fine forceps under a microscope.^27^ STs were cut to about 3‐cm‐long fragments and distributed randomly into 12‐well plates, with each well containing equal amount of ST fragments. The STs were cultured at 34°C and 5% CO_2_ for up to 14 days in LDM for induction of SLCs into ALCs to produce T as previously described.^27^, ^30^ Twelve isolations were performed. To study the effects of EGF on the proliferation of SLCs on the surface of STs, EGF (0, 1, and 10 ng/mL) was added to M199 medium and cultured at 34°C and 5% CO_2_ for 7 days, during which SLCs have the highest capacity of proliferation.^30^

### 2.5 EdU incorporation into SLCs

The proliferative capacity of SLCs after EGF treatment was measured by EdU kit as previously described.^33^ Media containing EGF and its inhibitor were removed for both CD90^+^ SLCs and STs above, and 2 µL of 1:1000 diluted EdU was added in the well and incubated at 34°C and 5% CO_2_ for 24 hours. STs were washed twice with 500 µL phosphate‐buffered saline (PBS) containing 3% BSA. Fragments of STs were also brought down in 2% argarose gel after centrifugation at 750 *g*, and the cross sections were cut. CD90^+^ SLCs and ST cross sections were then fixed in 4% paraformaldehyde at room temperature for 30 minutes. The CD90^+^ SLCs and STs were washed and incubated with reaction solution in the dark for 45 minutes. CD90^+^ antibody was used for staining CD90^+^ SLCs in purified SLCs. DAPI served the counterstaining. Then, CD90^+^ SLCs and STs were washed again and mounted on slide for visualization under fluorescence microscope (Olympus) and images were captured. EdU‐positive cells (green fluorescence in cell nucleus) were counted and calculated by the total surface area of STs using the ImageProPlus 7.0 software (Media Cybernetics).

### 2.6 SLC differentiation

Our previous study has demonstrated that STs are cultured in LDM for 14 days, and SLCs on the surface of STs are capable of differentiating into ALCs, which robustly produce T.^30^ To study the effects of EGF on the differentiation of SLCs, EGF (0, 1, and 10 ng/mL) with or without EGF antagonist (erlotinib, E, 100 nmol/L) was added to LDM and cultured at 34°C and 5% CO_2_ for 14 days. Then, media containing EGF were removed for measurement of T levels. Fragments of STs were also brought down in 2% argarose gel after centrifugation at 750 *g*, and the cross sections were cut. ST cross sections were then fixed in 4% paraformaldehyde at room temperature for 30 minutes. The STs were stained immunohistochemically after incubating HSD11B1 antibody (for ALC biomarker) and smooth actin muscle (SMA for peritubular myoid cells) and then Alexa Fluor 488 (green colour for HSD11B1) or Fluor 594 (red colour for α‐SMA) for 1 hour. The sections were stained with DAPI for the nucleus of the cells. The slides were covered with 50% glycerol. The fluorescence was visualized under a fluorescent microscope (Olympus).

### 2.7 SLC proliferation

Our previous study has demonstrated that the STs are cultured during the first week of culture, and the number of SLCs is greatly amplified.^30^ Then, STs were switched to LDM for additional 7 days, the amplified SLCs could be differentiated into ALCs, and thus, the increased number of ALCs could contribute into the robust increase of T level in the medium.^30^ Using this approach, EGF (0, 1 and 10 ng/mL) with or without EGF antagonist (E, 100 nmol/L) was added to M199 and cultured at 34°C and 5% CO_2_ for 7 days, and then, STs were switched into LDM for additional 7 days. Media were collected for the measurement of T level.

### 2.8 Isolation of PLCs

The procedure for PLC isolation and cultured was performed as previously described.^9^ Briefly, forty 21‐day‐old rats were euthanized under CO_2_ for isolation of PLCs. All testes were taken out and decapsulated and put in sterile 50‐mL tube (10 mL medium). Testes were digested in medium 199 containing 0.25 mg/mL collagenase‐D at 34°C in the shaking bath (75 rpm) for 10 minutes. The testis fragments were gently shaken and filtered through two layers of nylon mesh (200 μm) and washed with medium 199. The filtered cells were centrifuged at 250 *g* for 10 minutes. Crude cell preparations were resuspended in the 55% isotonic Percoll. Following density gradient centrifugation at 25 000 *g* at 4°C for 45 minutes, the PLC fraction was gently collected between densities of 1.064 and 1.070 g/mL. The cells were washed with HBSS and centrifuged at 250 *g* for 10 minutes. PLCs were resuspended in phenol red‐free 1:1 DMEM: F12 supplemented with 1 mg/mL BSA. Purity of PLCs was judged after histochemical staining of HSD3B1 activity with 0.4 mmol/L etiocholanolone and 0.4 mmol/L NAD^+^ as previously described.^34^ The purity of PLCs was typically more than 95%. The purifications of PLCs were repeated for four times.

### 2.9 [^3^H]‐Thymidine incorporation into PLCs

[^3^H]‐Thymidine incorporation into PLCs was used to assess cell proliferation as previously described.^17^ 1 × 10^6^ PLCs were cultured with DMEM: F12 (1:1) alone or in combination with 1 and 10 ng/mL EGF at 34°C 5% CO_2_ for 24 hours. Cells were incorporated with [^3^H]‐thymidine at 1 µCi/mL during the last 24 hours of incubation at 34°C. After the incorporation, PLCs were washed twice with PBS and harvested. PLCs were lysed in 0.5 mL hyamine hydroxide, and radioactivity was measured in a liquid scintillation counter (PE, USA). Cpm per 10^6^ PLCs was calculated for thymidine incorporation into PLCs.

### 2.10 Measurement of cellular H_2_O_2_‐induced reactive oxygen species in PLCs

Reactive oxygen species (ROS) production was measured with the fluorescence dye 2′7′‐dichlorofluorescin diacetate (DCFH‐DA) assay kit (Qcbio Science and Technologies Co., Ltd.). Briefly, 1.5 × 10^5^ cells/mL isolated PLCs were plated into the 6‐well plates and incubated for 24 hours. Then, cells were divided into four groups: control, 10 ng/mL EGF, 200 μmol/L H_2_O_2_, and 10 ng/mL EGF + 200 μmol/L H_2_O_2_. 200 μmol/L H_2_O_2_ was used as a positive inducer of ROS. Cells were cultured for 48 hours. Thereafter, cells were harvested and suspended with 200 μL DCFH‐DA for 20 minutes at 37°C in the dark. Cells were washed twice with PBS, and fluorescence intensity determined by flow cytometer was used to measure ROS.

### 2.11 Annexin V and PI assay for apoptosis of PLCs

Isolated PLCs were planted into the 6‐well plates with the density of 2.5 × 10^6^ cells/mL and incubated for 24 hours. Cells were divided into four groups: control, 10 ng/mL EGF, 200 μmol/L H_2_O_2_, and 10 ng/mL EGF + 200 μmol/L H_2_O_2_. 200 μmol/L H_2_O_2_ was used as a positive inducer of cell apoptosis. Cells were cultured for 48 hours. To evaluate early and lately apoptotic activity, an Annexin V‐FITC/PI Apoptosis Detection Kit (Nanjing KeyGEN Biotech) was used as previously described.^35^ Cells were harvested and washed with cold PBS and then were resuspended in 200 μL the annexin V‐binding buffer. After cells were stained with FITC‐labelled annexin V and PI, they were instantly measured using flow cytometer.

### 2.12 PLC steroidogenesis after EGF treatment

Progenitor Leydig cells with a density of 0.5 × 10^6^ cells per cell were cultured with DMEM: F12 (1:1) alone or in combination with 1 and 10 ng/mL EGF at 34°C 5% CO_2_ for 24 hours. Media were collected for measurement of AO and T. PLCs were washed twice with PBS and harvested for isolation of RNAs and proteins.

### 2.13 Medium T and androsterone analysis

Medium concentrations of T and AO were measured by the tritium‐based radioimmunoassay validated for the use of rat antiserum as using either anti‐T antibody (Fitzgerald, MA) or anti‐AO antibody.^9^ Standards ranging between 10 and 2000 pg/mL T or AO were prepared in triplicate. Standards and samples were incubated with respective tracer and antibody at 4°C overnight, and charcoal‐dextran suspension was used to separate the bound and free steroids. The bound steroids were mixed with a scintillation buffer and counted in a β‐scintillation counter (PE, USA). The minimum detectable concentration of the assay for either T or AO was 5 pg/mL. The quality control had either 100 pg/mL T or 100 pg/mL AO dissolved in the same culture media. Interassay and intra‐assay coefficients of variation for T and AO were within 10%.

### 2.14 Microarray hybridization and scanning

Progenitor Leydig cells were treated with 0, 1 and 10 ng/mL EGF as well as 1 ng/mL LH. LH serves the positive control for induction of PLC proliferation and differentiation. Total RNAs were harvested from PLCs after EGF treatment using a Trizol kit (Invitrogen) for microarray analysis. The RatRef‐12 Expression BeadChip containing 21 910 rat genes was used as previously described.^36^ Genes are selected from the NCBI RefSeq database to cover the whole rat transcriptome. Four groups of samples were used: 0, 1 and 10 ng/mL EGF‐treated as well as 1 ng/mL LH‐treated PLCs. Four replicates per group were performed. Probe labelling, hybridization, washing and scanning were performed using the Illumina Total Prep Kit (Applied Biosystems) as previously described.^37^ First strand of cDNA was synthesized in a total volume of 20 μL with the supplied reagents. The first‐strand product was used for the second‐strand synthesis, followed by column purification. The purified product was then used for in vitro transcription using T7 polymerase. Biotin‐16‐dUTP was incorporated and the biotinylated complementary RNA (cRNA) probe as prepared. The probe integrity was verified using the Agilent 2100 Bioanalyzer. Labelled cRNA (750 ng) was hybridized to the array chip overnight at 58°C in a total volume of 30 μL of hybridization buffer, followed by post‐hybridization stringency washing. The chip was scanned in the NextSeq 550 System (Illumina).

### 2.15 Microarray data analysis

Microarray data analysis was performed as previously described.^36^ Briefly, after scanning, the microarray data were imported into the BeadStudio software (Illumina) for normalization, preliminary analysis and filtering. The background subtraction was performed, and the Illumina custom error model was used to generate present/absent calls for each probe (“present” defined as *P* < .01 for signal detection) and to call differentially expressed genes (defined as *P* < .05 after false discovery rate correction). For each array, all probe sets were normalized to a mean signal intensity value of 100. Normalized data from BeadStudio were filtered to exclude genes not expressed in PLCs (ie data from probes that were classed as “absent” in all samples). All of the 21 910 genes were present in the data based on which further analyses were carried out. The data were further imported into Microsoft Access 2010, and queries to find the increased and decreased genes after EGF treatment when compared to the control were generated to find the expression levels.

### 2.16 Biological pathway analysis

Biological pathway analysis was performed as previously described.^36^ The Gene MicroArray Pathway Profiler 2.1 (GenMAPP2.1) software was used to find the biological pathway, and GO pathway was generated according to the software developer's instruction. The GenMAPP2.1 was used to create a map of signal pathways for the potential pathways. We imported our statistical results into the program and illustrated biological pathways containing differentially expressed genes. The results of the differential gene expression profile were validated by RT‐qPCR.

### 2.17 Quantitative real‐time PCR (RT‐qPCR)

Briefly, first‐strand synthesis of DNA and RT‐qPCR were performed as previously described.^38^ qPCR was carried out in a 20 µl volume in a 96‐well plate using the SYBR Green PCR Kit from Applied Biosystems. Primer titration was performed with the concentration of 300 nmol/L. Fluorescence was detected using the ABI 7700 system (PE Applied Biosystems). Each sample was run in duplicate and in parallel with no template controls. The relative mRNA levels of targeted genes were adjusted to housekeeping gene, ribosomal protein S16 (*Rps16*), as the internal control. *Rsp16* in LCs has been used as the internal control in many studies because it showed consistent expression.^32^, ^39^ The Ct value was read, and the levels of the target mRNAs were calculated using the standard curve method as previously described.^36^ All primers in the present study were designed by Primer 3 software (Whitehead Institute for Biomedical Research). Forward and reverse primers were placed in different exons to minimize the effects of possible DNA contamination. These genes are as follows: luteinizing hormone receptor (*Lhcgr*), scavenger receptor class B member 1 (*Scarb1*), *Star*, *Cyp11a1*, *Hsd3b1*, *Cyp17a1*, *Hsd17b3*, *Srd5a1*, *Akr1c9*, insulin‐like 3 (*Insl3*)*,* sulfotransferase 1A1 (*Sult1a1)* and cyclin D1 (*Ccnd1*). The primers were listed in Table S1.

### 2.18 Western blotting

Progenitor Leydig cells were homogenized and lysed. Protein concentrations in samples were measured using the Bio‐Rad Protein Assay Kit (Bio‐Rad) as previously described.^33^ BSA was used as the protein standard. Samples (50 µg protein) were boiled in equal volumes of sample loading buffer, containing 20% glycerol, 5% sodium dodecyl sulfate, 3.1% dithiothreitol and 0.001% bromophenol blue. Samples were electrophoresed on 10% polyacrylamide gels containing sodium dodecyl sulfate. Proteins were electrophoretically transferred onto nitrocellulose membrane. After one‐hour exposure to 5% non‐fat milk to block nonspecific binding, the membranes were incubated with the primary antibodies (1:1000 dilution) against LHCGR (Santa Cruz), CYP11A1 (Santa Cruz, Santa Cruz, CA), CYP17A1 (Santa Cruz), StAR (Pterosaur Biotech) and actin β (ACTB, Cell Signaling Technology). The membranes were then washed and incubated with a 1:1000 dilution of anti‐rabbit or anti‐goat antiserum (R&D Systems, Inc) that was conjugated to horseradish peroxidase. The washing step was repeated, and immunoreactive bands were visualized by chemiluminescence using an ECL kit (Amersham). The density was scanned by ImageJ software.

### 2.19 Statistics

Data were subjected to analysis by Student's *t* test to identify significant differences whenever two groups (a single concentration of EGF vs. control) were compared. Data were subjected to analysis by one‐way ANOVA test followed by ad hoc Tukey multiple comparisons to identify significant differences between the tested group and the controls whenever there were three or more groups (multiple concentrations of etomidate vs control) were compared. All data are expressed as means ± SEM. Differences were regarded as significant at *P* < .05.

## 3 RESULTS

### 3.1 EGF increases EdU incorporation into SLCs

**FIGURE 1:**
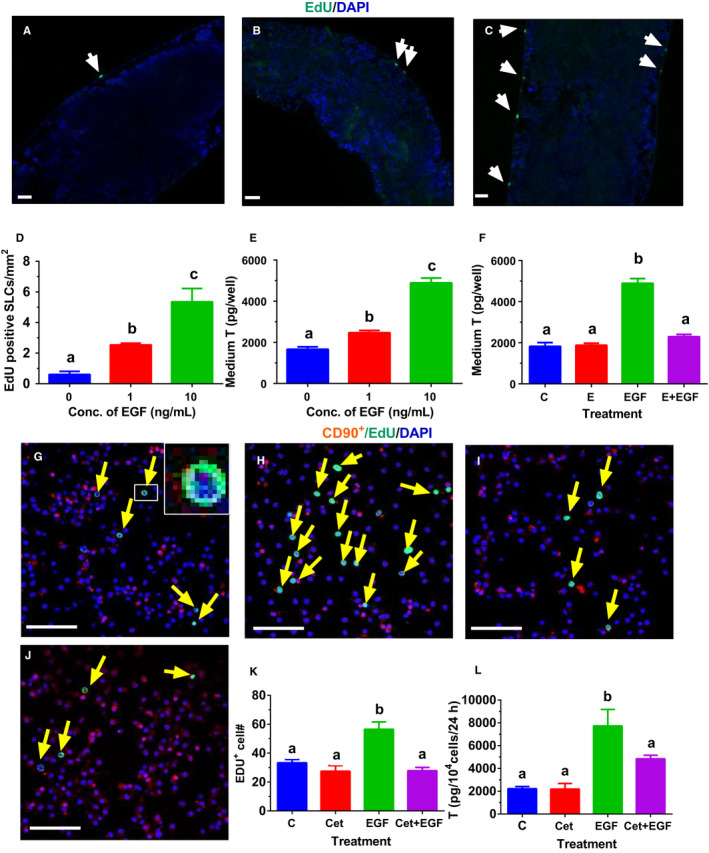
Effects of EGF on proliferation of stem Leydig cells (SLCs). Leydig cell (LC)‐depleted seminiferous tubules (STs) were cultured without (Panel A) or with 1 ng/mL EGF (Panel B), or 10 ng/mL EGF (Panel C) for 7 d. CD90^+^ SLCs were cultured without (Panel G) or with 10 ng/mL EGF (Panel H), or 5 μg/mL Cet (cetuximab, Panel I), or 10 ng/mL EGF + 5 μg/mL Cet (Panel J) for 24 h. EdU incorporation into the nucleus of SLC on the STs (white arrow) or CD90^+^ SLC (yellow arrow) was observed. DAPI serves as the counterstaining. EdU‐positive cells were located outside of STs. Bar = 50 μm. The EdU‐positive SLCs per mm^2^ STs were presented in Panel D (mean ± SEM, n = 5). In Panel E, STs were cultured without or with 1 or 10 ng/mL EGF for 7 d, and then, STs were switched to Leydig cell differentiation medium (LDM) for additional 7 d to produce testosterone (T), and the effects of EGF on SLC proliferation were indirectly analysed (mean ± SEM, n = 4). In Panel F, STs were treated with 0 ng/kg EGF (control, C) or 100 nmol/L EGF antagonist (erlotinib, E), or 10 ng/mL EGF (EGF) or 100 nmol/L E plus 10 ng/mL EGF (E + EGF) for 7 d, and then, STs were switched to LDM for 14 d to produce T (mean ± SEM, n = 4). The EdU‐positive CD90^+^ SLCs were presented in Panel K (mean ± SEM, n = 6). In Panel L, CD90^+^ SLCs were treated with 0 ng/kg EGF (control, C) or 5 μg/mL EGF antagonist (Cet), or 10 ng/mL EGF (EGF) or 5 μg/mL Cet plus 10 ng/mL EGF (Cet + EGF) for 7 d, and then, cells were switched to LDM for 7 d to produce T (mean ± SEM, n = 4). Identical letters designate no significant difference between two groups at *P* < .05

To investigate the effects of EGF on SLC proliferation, we treated STs for 7 days with EGF and/or EGF inhibitor in vitro. SLCs reside on the surface of the ST (Figure 1A‐C).^30^ EGF concentration‐dependently increased EdU incorporation into SLCs (Figure 1D). This indicates that EGF stimulates SLC proliferation. Our previous study has shown that during the first week SLCs had the highest proliferative capacity and LDM was capable of inducing the differentiation of SLCs into ALCs, which robustly produced T after additional 7 days in LDM.^30^ Using this indirect approach, we treated STs with 0‐10 ng/mL EGF for 7 days and then switched STs in LDM for additional 7 days to induce the formation of ALCs, which produced T. As shown in Figure 1E, EGF concentration‐dependently increased medium T levels after 7‐day culture, indicating that EGF is capable of increasing the number of SLCs that are differentiated into ALCs, which produce more T. We also tested EGF action using EGF antagonist (Erlotinib, E). Indeed, E (100 nmol/L) alone did not affect T level but it can reverse EGF (10 ng/mL)‐induced action (Figure 1F). This indicates that EGF acts via EGF receptor. In order to test the effects of EGF on SLC proliferation, purified CD90^+^ SLCs were treated with EGF and/or its antagonist (Cetuximab, Cet) for 24 hours. EGF (10 ng/mL) significantly increased EdU incorporation into CD90^+^ SLCs (Figure 1H,K) when compared to the control (Figure 1G). Cet (5 µg/mL, Figure 1I) alone did not affect EdU incorporation but it can reverse EGF (10 ng/mL)‐induced action (Figure 1J,K). Using the indirect approach, we treated CD90^+^ SLCs with 0‐10 ng/mL EGF for 7 days and then switched CD90^+^ SLCs in LDM for additional 7 days to induce the formation of ALCs. As shown in Figure 1L, EGF increased medium T levels after 7‐d culture, indicating that EGF is capable of increasing the number of SLCs that are differentiated into ALCs. This further confirms that EGF acts via EGF receptor to stimulate SLC proliferation.

### 3.2 EGF inhibits differentiation of SLCs into the LC lineage

**FIGURE 2:**
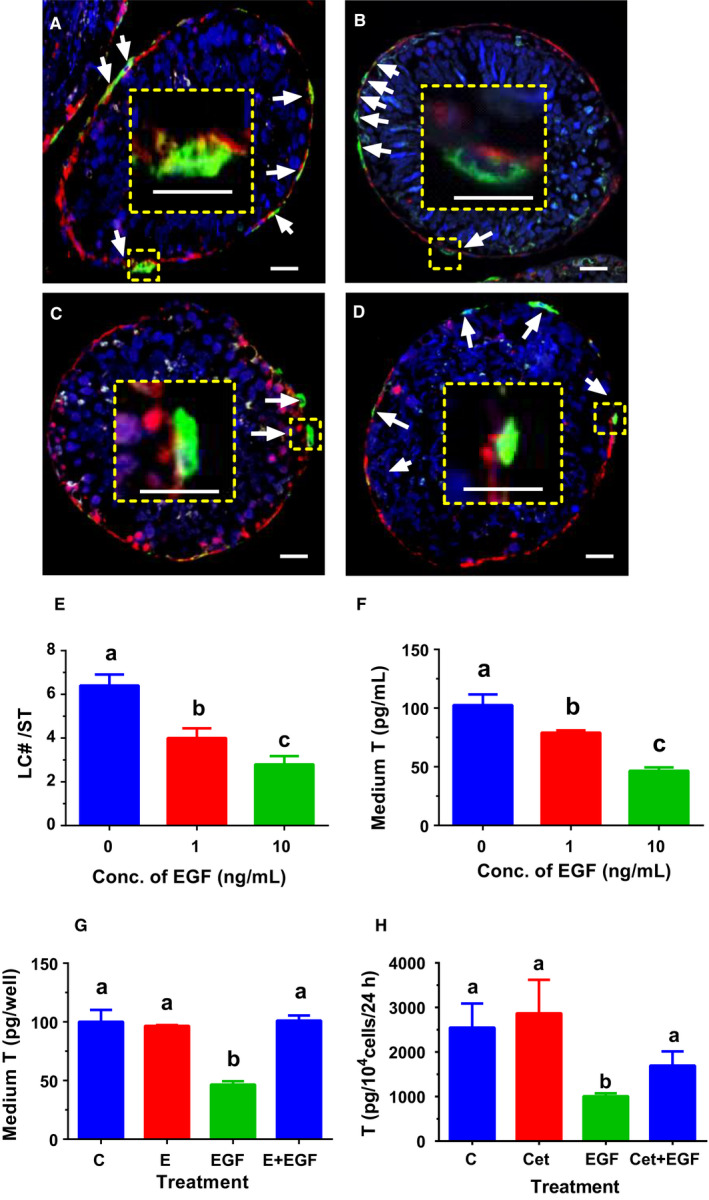
Effects of EGF on differentiation of stem Leydig cells (SLCs) in vitro. Leydig cell (LC)‐depleted seminiferous tubules (STs) were cultured LC differentiation medium (LDM) containing EGF (0 ng/mL, Panel A), EGF antagonist (erlotinib, E, 1 µmol/L, Panel B), EGF (10 ng/mL, Panel C) and EGF (10 ng/mL) + E (1 µmol/L, Panel D) for 14 d. LCs were identified by biomarker 11β‐hydroxysteroid dehydrogenase 1 (HSD11B1, green colour in the cytoplasm, white arrow). Peritubular myoid cells were stained by smooth muscle actin (red colour in the cytoplasm), drawing a boundary for the ST. DAPI serves as counterstaining. Inset is the magnified picture, showing that HSD11B1 positive LCs existed outside of STs. Bar = 10 μm. The HSD11B1‐positive LCs per ST in the cross section were presented in Panel E (mean ± SEM, n = 4). In Panel F, STs were cultured in LDM without or with 1 or 10 ng/mL EGF for 14 d, and medium T levels were measured (mean ± SEM, n = 4). In Panel G, STs were treated in LDM with 0 ng/kg EGF (control, C) or 100 nmol/L EGF antagonist (erlotinib, E), or 10 ng/mL EGF (EGF) or 100 nmol/L E plus 10 ng/mL EGF (E + EGF) for 14 d, and medium T levels were measured (mean ± SEM, n = 4). In Panel H, CD90^+^ SLCs were treated with 0 ng/kg EGF (control, C) or 5 μg/mL EGF antagonist (Cet), or 10 ng/mL EGF (EGF) or 5 μg/mL Cet plus 10 ng/mL EGF (Cet + EGF) for 14 d to produce T (mean ± SEM, n = 4). Identical letters designate no significant difference between two groups at *P* < .05

To examine whether EGF is capable of affecting SLC differentiation, STs were cultured in M199 for 7 days and then were switched to EGF (0, 1, and 10 ng/mL)‐containing LDM for additional 7 days. We used HSD11B1 as the biomarker of ALCs. After 7‐day culture in LDM, many HSD11B1 positive ALCs were formed on the surface of STs, designating ALCs (Figure 2A). EGF antagonist (E, 100 nmol/L) did not affect the number of LCs (Figure 2B). However, EGF (10 ng/mL) significantly reduced the number of HSD11B1 positive LCs, while EGF antagonist E reversed EGF‐mediated inhibition (Figure 2D). EGF concentration‐dependently lowered LC number per ST (Figure 2E) and medium T levels (Figure 2F). EGF antagonist E did not affect medium T level but reversed EGF (10 ng/mL)‐induced suppression of T synthesis (Figure 2G). We further cultured CD90^+^ SLCs in the presence of EGF (10 ng/mL) and/or Cet (5 μg/mL) for 14 days. EGF antagonist Cet did not affect medium T output but reversed EGF (10 ng/mL)‐induced suppression of T synthesis (Figure 2H). These data indicate that EGF inhibits SLC differentiation into the LC lineage via EGF receptor.

### 3.3 EGF inhibits differentiation of PLCs

**FIGURE 3:**
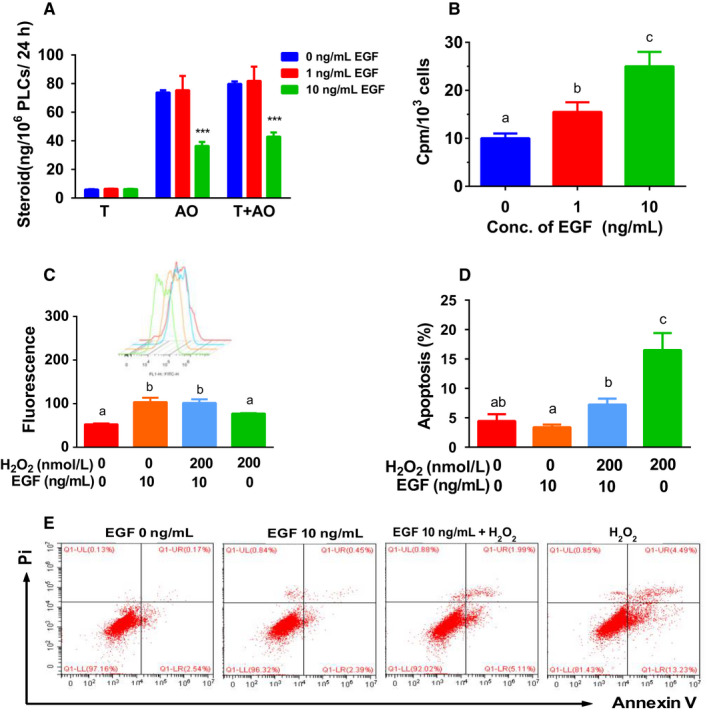
Effects of EGF on androgen production, proliferation, ROS generation and apoptosis by progenitor Leydig cells (PLCs) in vitro. Panel A, PLCs were cultured with 0, 1 and 10 ng/mL EGF for 24 h, and medium levels of testosterone (T) and androsterone (AO) were measured; mean ± SEM, n = 8. Panel B, PLCs were cultured with 0, 1 and 10 ng/mL EGF for 24 h, and PLCs were incorporated with [^3^H]‐thymidine for additional 24 h, and CPM was counted; mean ± SEM, n = 4. Panel C, PLCs were treated with control, 10 ng/mL EGF, 200 μmol/L H_2_O_2_ and 10 ng/mL EGF + 200 μmol/L H_2_O_2_ for 24 h, and ROS was counted, mean ± SEM, n = 3, inset is ROS fluorescence image; Panel D, PLCs were treated with control, 10 ng/mL EGF, 200 μmol/L H_2_O_2_ and 10 ng/mL EGF + 200 μmol/L H_2_O_2_ for 24 h, and apoptosis was counted; mean ± SEM, n = 4. Panel E, apoptosis image. Identical letters designate no significant difference between two groups at *P* < .05

To examine whether EGF can affect PLC differentiation, PLCs were cultured with EGF (0, 1, and 10 ng/mL) in DMEM: F12 medium for 48 hours. EGF (10 ng/mL) significantly lowered AO and total androgen (T plus AO) production by PLCs (Figure 3A). The data indicate that EGF inhibits PLC differentiation.

### 3.4 EGF increases thymidine incorporation into PLCs

To investigate the effects of EGF on PLC proliferation, we treated PLCs for 48 hours in vitro. Both concentrations of EGF significantly increased thymidine incorporation into PLCs (Figure 3B). This indicates that EGF stimulates PLC proliferation.

### 3.5 EGF and H_2_O_2_ induce ROS generation in PLCs

To investigate the effects of EGF on ROS generation by PLCs, we treated PLCs with EGF for 48 hours. H_2_O_2_ was used as a positive control (Figure 3C). Indeed, H_2_O_2_ (200 μmol/L) induced ROS generation in PLCs. Interestingly, EGF (10 ng/mL) also induced ROS generation in PLCs (Figure 3C). EGF plus H_2_O_2_ did not further increased ROS production in PLCs (Figure 3C). This indicates that EGF and H_2_O_2_ induce ROS production via different mechanisms.

### 3.6 EGF antagonizes H_2_O_2_‐induced PLC apoptosis

To investigate the effects of EGF on PLC apoptosis, we treated PLCs with EGF for 48 hours. H_2_O_2_ was used as the positive control to induce apoptosis (Figure 3D,E). Indeed, H_2_O_2_ induced PLC apoptosis at 200 μmol/L. EGF (10 ng/mL) did not affect PLC apoptosis. However, EGF antagonized H_2_O_2_‐induced PLC apoptosis (Figure 3D,E). This indicates that EGF protects PLCs from H_2_O_2_‐mediated apoptosis.

### 3.7 EGF‐mediated gene expression of PLCs

**FIGURE 4:**
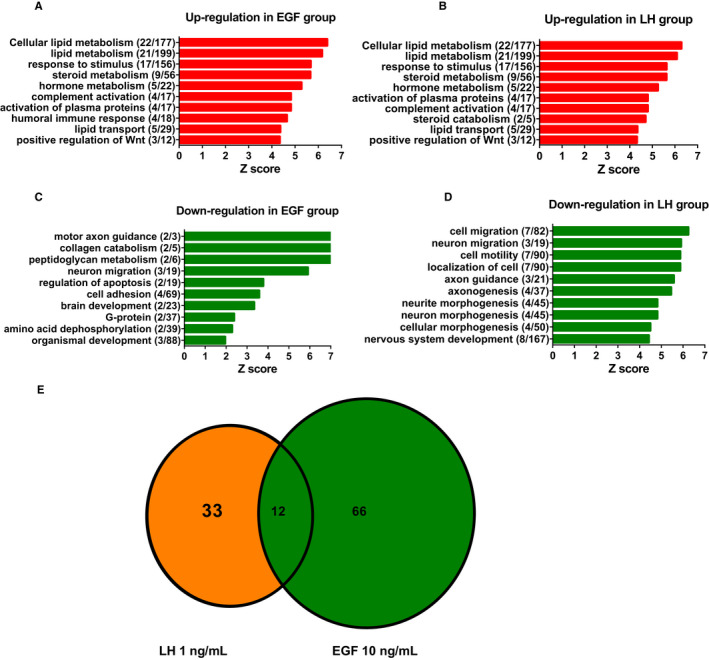
Effects of EGF and LH on gene expression in progenitor Leydig cells: GO analysis. After rat progenitor Leydig cells (PLCs) were cultured with 0, 1 and 10 ng/mL EGF as well as 1 ng/mL LH for 24 h, cells were subjected to gene microarray analysis. Panels A and B: up‐regulation by EGF and LH, respectively; Panels C and D: down‐regulation by EGF and LH, respectively; and Panel E: overlapping genes in EGF or LH up‐regulated genes

Luteinizing hormone is an important hormone for PLC development.^2^ We used LH (1 ng/mL) as the positive control. PLCs were cultured with EGF (0, 1 and 10 ng/mL) in DMEM: F12 medium for 24 hours. Microarray of gene profiles of PLCs after EGF treatment were compared to LH (1 ng/mL). A genome‐wide expression containing 21 910 probes was analysed. Of these probes, 8667 probes were detected in the control group. Of them, 33 genes were up‐regulated and 53 genes down‐regulated more than twofold in 1 ng/mL LH control group. Eighteen genes were up‐regulated (Table 1) and 81 genes down‐regulated (Table 2) more than twofold in 1 ng/mL EGF group, while 66 genes were up‐regulated (Table 1) and 198 genes down‐regulated (Table 2) more than twofold in 10 ng/mL EGF group. GO analysis showed that up‐regulation of genes by EGF (10 ng/mL) includes the following 10 major categories: lipid metabolism, response to stimulus, steroid metabolism, hormone metabolic process, complement activation, activation of plasma proteins, humoral immune response, lipid transport, positive regulation of Wnt, axon guidance, response to nutrient, and immune response (Figure 4A). The down‐regulation of genes by EGF (10 ng/mL) includes the following 10 major categories: motor axon guidance, collagen catabolism, peptidoglycan metabolism, neuron migration, regulation of apoptosis, cell adhesion, brain development, G‐protein, amino acid dephosphorylation and organismal development (Figure 4C). When compared to LH (1 ng/mL), most of categories up‐regulated by EGF (10 ng/mL) were overlapped with those of LH (Figure 4B), except one (humoral immune response) for EGF and one (steroid catabolism) for LH. Of 33 up‐regulated genes by LH, 12 genes were also up‐regulated by EGF (Figure 4E). When compared to LH, the categories down‐regulated by EGF were more diversified (Figure 4D).

**TABLE 1:** Genes were up‐regulated after EGF treatment

Gene	Gene name	Expression levels				
		EGF (ng/mL)			LH (ng/mL)	
		0	1	10	1	
Cell cycle regulation/ DNA repair						
Ercc1	Endonuclease non‐catalytic subunit	641 ± 35	1138 ± 30 (1.8)	1750 ± 115 (+2.7)		951 ± 41 (+1.5)
Ccnd1	Cyclin D1	85 ± 3	120 ± 2 (+1.4)	219 ± 1 (+2.6)		93 ± 3 (+1.1)
Phlda11	Pleckstrin homology like domain A	246 ± 11	438 ± 17 (+1.8)	621 ± 3 (+2.5)		528 ± 2 (+2.2)
Reln	Reelin	415 ± 6	643 ± 26 (+1.5)	1038 ± 43 (+2.5)		819 ± 29 (+2.0)
Schip1	Schwannomin interacting protein 1	140 ± 2	245 ± 8 (+1.8)	319 ± 6 (+2.3)		211 ± 5 (+0.6)
Cd82	Metastasis suppressor kangai‐1	478 ± 6	785 ± 14 (+1.6)	1070 ± 28 (+2.2)		754 ± 16 (+1.6)
Cell metabolism						
Upp1	Uridine phosphorylase 1	1006 ± 54	3816 ± 41 (+3.8)	5182 ± 99 (+5.1)		2897 ± 38 (+2.8)
Car2	Carbonic anhydrase II	250 ± 3	721 ± 29 (+2.9)	1093 ± 72 (+4.4)		1033 ± 20 (+4.2)
Hs3st1	heparan sulfate‐glucosamine 3‐sulfotransferase 1	146 ± 14	393 ± 5 (+2.7)	577 ± 14 (+4.0)		275 ± 8 (+2.0)
Dusp6	Dual specificity phosphatase 6	1150 ± 25	3183 ± 195 (+2.8)	4408 ± 81 (+3.8)		202 848± (+1.9)
Aadat	Aminoadipate aminotransferase	304 ± 4	763 ± 31 (+2.5)	971 ± 15 (+3.2)		1963 ± 36 (+6.2)
Smpd1	Sphingomyelin phosphodiesterase 1	6626 ± 242	14 384± (+2.2)	18 775± (+2.8)		12 677 ± 807 (+2.0)
Plscr1	Phospholipid scramblase 1	166 ± 6	280 ± 25 (+1.7)	423 ± 28 (+2.6)		459 ± 4 (2.7)
Lipg	Endothelial lipase	145 ± 1	254 ± 13 (+1.8)	337 ± 24 (+2.3)		201 ± 3 (+0.6)
Ligand/receptors						
Adm	Adrenomedullin	1662 ± 46	3557 ± 71 (+2.1)	4828 ± 68 (+2.9)		2131 ± 51 (+1.2)
Gal	Galanin	542 ± 23	1229 ± 48 (+2.3)	1524 ± 100 (+2.8)		558 ± 28 (+1.1)
Gchfr	GTP cyclohydrolase I	175 ± 8	347 ± 3 (+2.0)	465 ± 12 (+2.7)		150 ± 10 (+1.0)
Serpine1	Plasminogen activator inhibitor 1	805 ± 41	1819 ± 66 (+2.3)	2124 ± 29 (+2.6)		1482 ± 29 (+1.6)
Plcd4	Phospholipase C delta 4	422 ± 13	1014 ± 23 (+2.4)	1081 ± 24 (+2.6)		447 ± 24 (+1.0)
Tnfrsf12a	TNF receptor superfamily 12A	1230 ± 17	2237 ± 88 (+1.8)	3146 ± 120 (+2.6)		2189 ± 69 (+1.6)
Cxcr4	C‐X‐C motif chemokine receptor 4	253 ± 9	491 ± 19 (+1.9)	626 ± 11 (+2.5)		444 ± 23 (+1.6)
Mmp10	Matrix metalloproteinases 10	3312 ± 113	5671 ± 135 (+1.7)	12 386 ± 255 (+3.7)		4322 ± 103 (+1.3)
Alcam	CD166 antigen	560 ± 7	971 ± 27 (+1.7)	1367 ± 51 (+2.4)		641 ± 25 (+1.3)
Procr	Protein C receptor	466 ± 17	712 ± 10 (+1.5)	1097 ± 96 (+2.4)		1088 ± 38 (+2.0)
Ednrb	Endothelin receptor type B	2304 ± 73	4360 ± 61 (+1.9)	5238 ± 232 (+2.3)		7117 ± 278 (+3.0)
Areg	Amphiregulin	238 ± 9	385 ± 18 (+1.6)	488 ± 4 (+2.2)		406 ± 22 (+1.8)
Pdgfa	Platelet‐derived growth factor A	1734 ± 37	2475 ± 84 (+1.4)	3736 ± 43 (+2.2)		2530 ± 81 (+1.4)
Stc1	Stanniocalcin 1	1818 ± 86	2899 ± 92 (+1.6)	3809 ± 110 (+2.1)		3773 ± 265 (+2.0)
Transcription factor activity						
Maf	Proto‐oncogene c‐Maf	697 ± 17	2198 ± 68 (+3.2)	2914 ± 55 (+4.2)	1420 ± 15 (+2.2)	
Hmga1	High mobility group AT‐hook 1	370 ± 11	812 ± 1 (+2.2)	1011 ± 57 (+2.7)	662 ± 38 (+1.8)	
Ehd4	EH domain containing 4	459 ± 17	820 ± 50 (+1.8)	1123 ± 62 (+2.4)	710 ± 29 (+1.6)	
Runx1	Runt related transcription factor 1	607 ± 25	1276 ± 40 (+2.1)	1476 ± 59 (+2.4)	1542 ± 65 (+2.5)	
Arhgap22	Rho GTPase activating protein 22	148 ± 4	259 ± 1 (+1.7)	349 ± 10 (+2.4)	194 ± 16 (+1.3)	
Abhd3	Abhydrolase domain containing 3	980 ± 34	1876 ± 60 (+1.9)	2092 ± 164 (+2.1)	1608 ± 56 (+1.6)	
Twist1	Twist BHLH transcription factor	275 ± 13	464 ± 8 (+1.7)	587 ± 46 (+2.1)	434 ± 3 (+1.5)	
Transporter/Protein binding						
Slc17a1	solute carrier family 1 member 17a1	510 ± 3	1423 ± 27 (+2.8)	1845 ± 70 (+3.6)	569 ± 48 (+1.0)	
Slc1a5	solute carrier family 1 member 5	935 ± 38	2153 ± 41 (+2.3)	3114 ± 35 (+3.3)	1663 ± 26 (+1.7)	
Slc16a3	Solute carrier family 16 member 3	1858 ± 23	3451 ± 207 (+1.9)	4645 ± 250 (+2.5)	5015 ± 284 (+2.8)	
Igfbp3	IGF‐1 binding protein 3	589 ± 33	905 ± 33 (+1.5)	1400 ± 30 (+2.4)	917 ± 48 (+1.2)	
Sh2b3	SH2B adaptor protein 3	382 ± 16	727 ± 9 (+1.9)	785 ± 18 (+2.2)	520 ± 11 (+1.3)	
Ak3l1	Adenylate kinase isoenzyme 4	462 ± 28	885 ± 24 (+1.9)	1004 ± 31 (+2.2)	1089 ± 16 (+2.1)	
Ube2q2	Ubiquitin conjugating enzyme E2Q2	518 ± 10	875 ± 23 (+1.7)	1125 ± 45 (+2.2)	720 ± 16 (+1.4)	

Note

() fold up‐regulated when compared to control.

**TABLE 2:** Genes were down‐regulated after EGF treatment

Gene symbol		Expression level			
		EGF (ng/mL)			LH (ng/mL)
		0	1	10	1
Cell metabolism					
Hmgcs2	3hydroxy3methylglutarylCoA synthase 2	5387 ± 119	867 ± 52 (−6.2)	454 ± 15 (−11.9)	1019 ± 33 (−5.3)
Aldh1a1	Aldehyde dehydrogenase 1A1	18 447 ± 563	4873 ± 165 (−3.8)	1872 ± 103 (−9.9)	2162 ± 185 (−8.6)
Adh1	Alcohol dehydrogenase 1	1927 ± 52	616 ± 11 (−3.1)	397 ± 6 (−4.9)	558 ± 34 (−3.4)
Srpx	Sushi repeat‐containing protein	1356 ± 44	458 ± 22 (−3.0)	284 ± 6 (−4.8)	920 ± 40 (−1.5)
Es1	ES1 protein	2088 ± 23	804 ± 22 (−2.6)	442 ± 31 (−4.7)	908 ± 51 (−2.3)
Ddah2	Dimethylarginine dimethylaminohydrolase2	4845 ± 117	1906 ± 63 (−2.5)	1205 ± 16 (−4.0)	2512 ± 61 (−1.9)
Aox1	Aldehyde oxidase 1	6698 ± 140	2892 ± 137 (−2.3)	1724 ± 77 (−3.9)	2316 ± 57 (−2.9)
Ces3	Carboxylesterase 3	515 ± 19	174 ± 6 (−3.0)	134 ± 1 (−3.9)	173 ± 5 (−3.0)
Ssg1	Steroid‐sensitive gene 1	1226 ± 37	389 ± 15 (−3.2)	321 ± 13 (−3.8)	517 ± 22 (−2.3)
Ldhb	Lactate dehydrogenase‐B	2753 ± 18	1153 ± 51 (−2.4)	795 ± 19 (−3.5)	1663 ± 98 (−1.7)
Cyp27a1	Cytochrome P450 family 27 A1	2436 ± 67	1046 ± 30 (−2.3)	716 ± 17 (−3.4)	1473 ± 55 (−1.6)
Dpep1	Dipeptidase 1	949 ± 9	395 ± 7 (−2.4)	296 ± 21 (−3.2)	459 ± 22 (−2.0)
Pah	Phenylalanine hydroxylase	335 ± 3	138 ± 3 (−2.4)	109 ± 1 (−3.1)	202 ± 1 (−1.7)
Gstm2	Glutathione S‐transferase mu 2	1611 ± 95	704 ± 30 (−2.3)	542 ± 16 (−3.0)	744 ± 67 (−2.1)
Ligand					
Sectm1a	Secreted and transmembrane protein 1A	1904±	470± (−4.0)	237± (−8.1)	832± (−2.3)
Sectm1	Secreted and transmembrane 1	820±	287± (−2.9)	143 (−5.9)	734± (−1.1)
Cx3cl1	C‐X3‐C motif chemokine ligand 1	4969±	1865± (−2.7)	892± (−5.6)	3050± (−1.6)
CD302	C‐type lectin domain family 13 A	3907±	1253± (−3.1)	745± (−5.2)	2476± (−1.6)
Akr1c9	3α‐Hydroxysteroid dehydrogenase	960±	201± (−4.8)	128± (−7.5)	344± (−2.8)
Wnt6	Wnt6	1805±	501± (−3.6)	358± (−5.1)	414± (−4.4)
Wnt4	Wnt4	456±	148± (−3.1)	108± (−4.2)	217± (−2.1)
Cxcl12	C‐X‐C motif chemokine ligand 12	472±	147 ± (−3.2)	117± (−4.0)	215± (−2.0)
Ogn	Osteoglycin	3438±	1482 ± (−2.3)	1064 ± (−3.2)	1313± (−2.6)
Mdk	Neurite growth‐promoting factor 2	1107±	492 ± (−2.3)	355 ± (−3.1)	683± (−1.6)
Plxdc2	Plexin domain containing 2	5029±	2348± (−2.3)	1659± (−3.1)	3075 ± (−1.6)
Ly6e	Lymphocyte antigen 6 family member E	2887±	1371± (−2.1)	957± (−3.1)	1463± (−2.0)
Protein binding					
Selenbp1	Selenium binding protein 1	7799 ± 275	2319 ± 144 (−3)	1323 ± 75 (−5.9)	1757 ± 102 (−4.4)
Itm2a	Integral membrane protein 2A	4833 ± 235	1571 ± 40 (−3.1)	945 ± 79 (−5.1)	1469 ± 31 (−3.3)
Mx2	Interferon‐induced GTP‐binding protein	940 ± 30	442 ± 15 (−2.1)	262 ± 16 (−3.6)	519 ± 6 (−1.8)
Serping1	Serpin family G member 1	2185 ± 63	910 ± 12 (−2.4)	612 ± 9 (−3.5)	1384 ± 11 (−1.6)
C4‐2	MHC‐linked complement C4	487 ± 23	190 ± 5 (−2.6)	139 ± 1 (−3.5)	244 ± 6 (−2.0)
Rcan2	Regulator of calcineurin 2	385 ± 17	140 ± 1 (−2.8)	115 ± 5 (−3.4)	168 ± 8 (−2.2)
Lrrc17	Leucine rich repeat containing 17	1498 ± 32	630 ± 20 (−2.3)	448 ± 22 (−3.3)	571 ± 22 (−2.6)
Wbp5	Transcription elongation factor A9	3374 ± 102	1633 ± 28 (−2.1)	1105 ± 35 (−3.1)	1787 ± 145 (−1.8)
Serpina3n	Serpin family A member 3	1036 ± 25	514 ± 19 (−2.1)	343 ± 16 (−3.1)	844 ± 14 (−1.2)
Signal transduction					
Rhoj	Ras homolog family member J	1261 ± 51	511 ± 18 (−2.5)	378 ± 13 (−3.3)	546 ± 15 (−2.3)
Ngfrap11	Protein BEX3	1501 ± 54	672 ± 17 (−2.2)	458 ± 24 (−3.3)	789 ± 46 (−1.9)
Gas7	Growth arrest specific 7	1307 ± 12	598 ± 13 (−2.3)	429 ± 11 (−3.1)	638 ± 37 (−2.1)
C1qtnf7	C1q And TNF Related 7	1535 ± 45	640 ± 10 (−2.4)	347 ± 8 (−4.4)	807 ± 14 (−1.9)
Structure					
Col3a1	Collagen type III alpha 1	1469 ± 24	462 ± 22 (−3.2)	302 ± 19 (−4.9)	542 ± 33 (−2.7)
Apoe	Apolipoprotein E	8793 ± 489	3588 ± 44 (−2.5)	2711 ± 162 (−3.2)	4733 ± 183 (−2.9)
Epb4.1l3	Erythrocyte membrane protein band 4.1	602 ± 15	271 ± 7 (−2.1)	203 ± 6 (−3.0)	358 ± 11 (−1.8)

Note

() fold changes when compared to control.

### 3.8 PCR analysis of gene expression after EGF treatment

**FIGURE 5:**
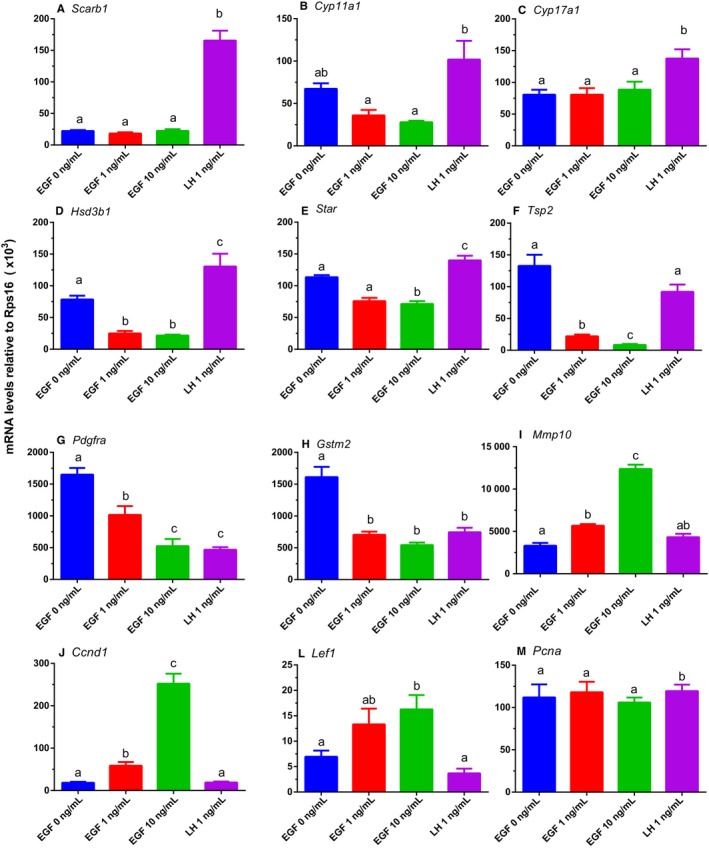
Effects of EGF on the expression levels of steroidogenesis‐related genes in rat immature Leydig cells. After rat progenitor Leydig cells (PLCs) were cultured with 0, 1 and 10 ng/mL EGF as well as 1 ng/mL LH for 24 h, cells were subjected to qPCR analysis. The expression levels of steroidogenesis‐related genes were measured and calculated adjusted to *Rps16*, the internal control. Mean ± SEM, n = 4; identical letters indicate that there is no significant difference between two groups at *P* < .05

We used PCR to analyse gene expression in EGF‐treated PLCs. We compared EGF with LH (Figure 5). The following patterns were shown: (a) no change in EGF but up‐regulation in LH (*Scarb1*, *Cyp11a1*, and *Cyp17a1*); (b) down‐regulation in EGF but up‐regulation in LH (*Star, Hsd3b1,* and *Tsp2*); (c) down‐regulation in both EGF and LH groups (*Pdgfra* and *Gstm2*); (d) up‐regulation in EGF but no change in LH (*Ccnd1, Mmp10,* and *Lef1*); and (e) no change in both EGF and LH groups (*Pcna*). PCR results were similar to that from microarray data.

### 3.9 Major pathways after EGF treatment

**FIGURE 6:**
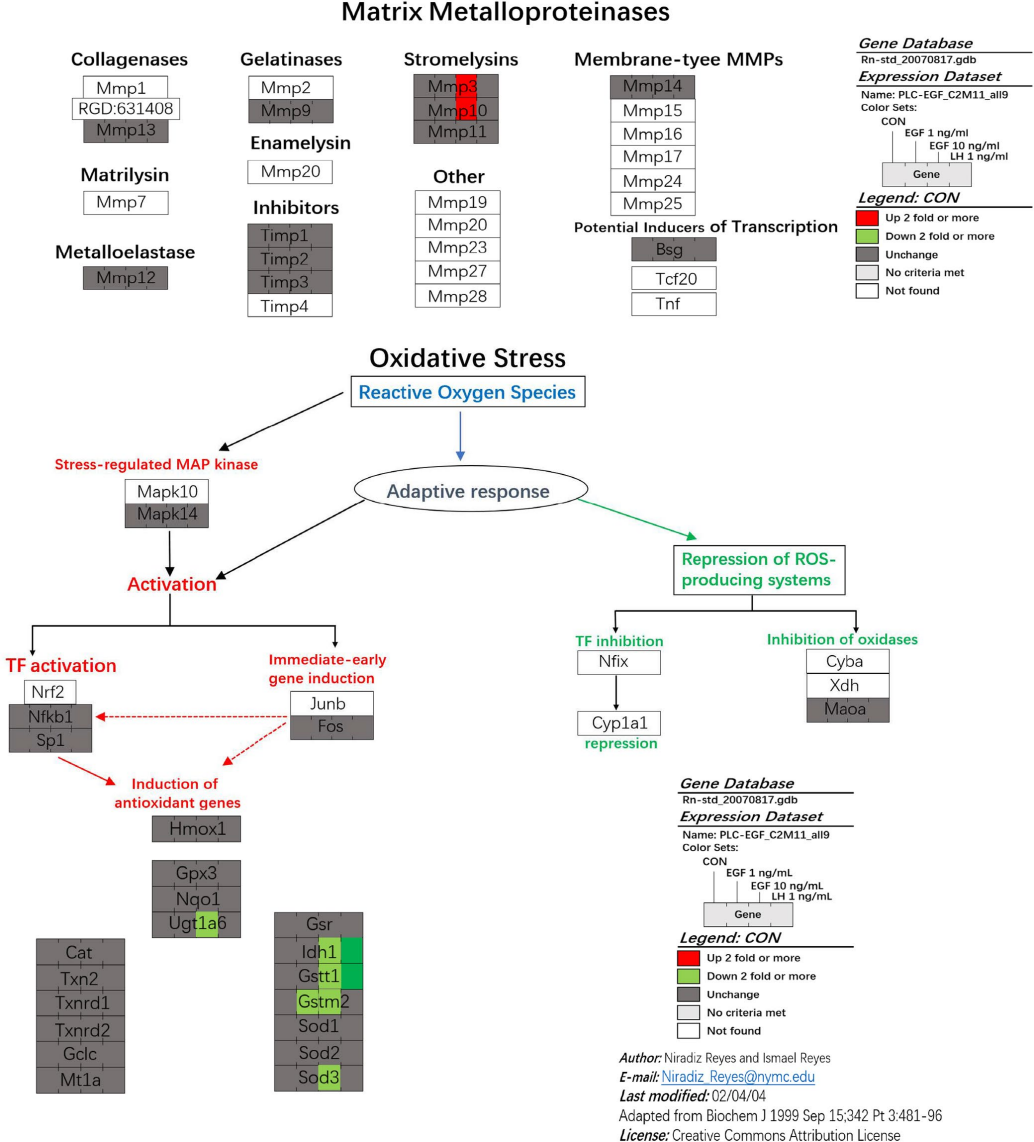
EGF up‐regulates some matrix metalloproteinase expression and down‐regulates some antioxidative protein expression in progenitor Leydig cells. After rat progenitor Leydig cells (PLCs) were cultured with 0, 1 and 10 ng/mL EGF as well as 1 ng/mL LH for 24 h, cells were subjected to gene microarray analysis. Expression of matrix metalloproteinases was analysed. Expression of antioxidative proteins was analysed

Using GenMAPP2, we discovered several pathways that were specific to the regulation of EGF. The expression of matrix metalloproteinases (*Mmp3* and *Mmp10*) was significantly up‐regulated by more than twofold by EGF (Figure 6). Interestingly, the antioxidant genes (*Ugt1a6*, *Idh1*, *Gsst1*, *Gstm2* and *Sod3*) were down‐regulated by EGF (Figure 6), indicating that EGF might lead to ROS accumulation. Of these 5 genes, two (*Gsst1* and *Gstm2*) were also down‐regulated by LH (Figure 6).

### 3.10 EGF lowers steroidogenesis‐related protein levels via EGFR signalling

**FIGURE 7:**
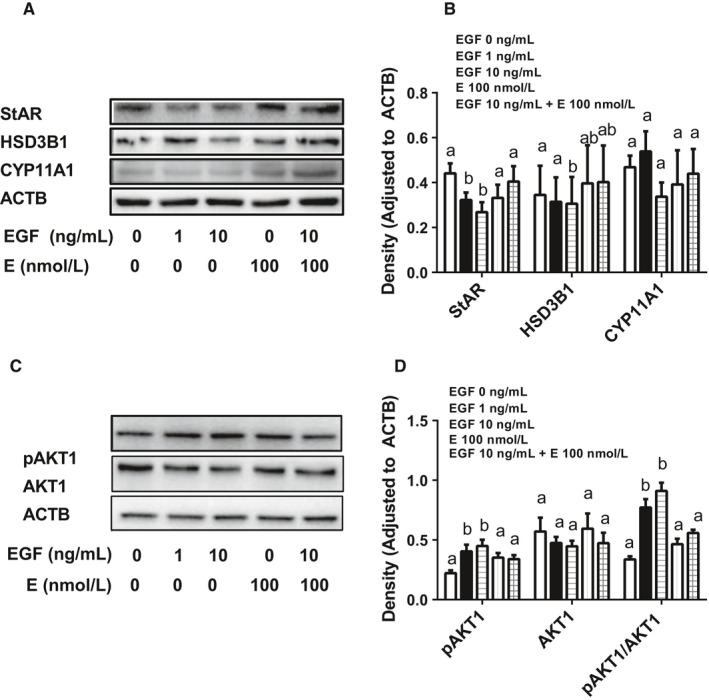
Effects of EGF on steroidogenesis‐related proteins and AKT1 phosphorylation in rat progenitor Leydig cells. After rat progenitor Leydig cells (PLCs) were cultured with 0, 1 and 10 ng/mL EGF as well as 1 ng/mL LH for 24 h, cells were subjected to Western blot. The levels of steroidogenesis‐related proteins (StAR, HSD3B1 and CYP11A1, Panels A and B) as well as AKT1 and its phosphorylated protein (Panel C and D) were analysed. Proteins were measured and calculated adjusted to β‐actin (ACTB), the internal control. Mean ± SEM, n = 4‐7; identical letters indicate that there is no significant difference between two groups at *P* < .05

We performed Western blot to identify changes of steroidogenesis‐related proteins (including StAR, HSD3B1 and CYP11A1) in PLCs after treatment with EGF and/or EGFR antagonist (E) for 24 hours. As shown in Figure 7A, EGF concentration‐dependently lowered StAR and HSD3B1 protein levels without affecting CYP11A1 and E can reverse the action of EGF (10 ng/mL). This confirms the EGF‐induced mRNA changes.

### 3.11 EGF lowers the phosphorylation of AKT1 signalling

We performed Western blot to identify the changes of AKT1 as well as its phosphorylated protein in PLCs after treatment with EGF and/or E for 24 hours. As shown in Figure 7B, EGF concentration‐dependently increased phosphorylation of AKT1 without affecting total AKT1 level (Figure 7B). This suggests that the phosphorylation of AKT1 is involved in EGFR pathway.

## 4 DISCUSSION

In the present study, we found that EGF stimulated SLC/PLC proliferation but blocked their differentiation.

There is a growing evidence to indicate that EGF is a critical proliferative growth factor for SLCs or PLCs during pubertal development.^13^, ^40^, ^41^, ^42^ Apparently, EGF stimulated SLC and PLC proliferation in two‐type LC precursor cells: SLCs on STs and CD90^+^ SLCs (Figure 1) and isolated PLCs (Figure 3). Previous studies have demonstrated that CD90^+^ cells on the surface of STs were SLCs and can be induced into ALCs in LDM in vitro.^30^ CD90^+^ SLCs also contain other stem cell biomarkers including nestin, Cd51, Coup‐tf2, Arx, *Pdgfra* and Tcf21.^43^ This indicated that EGF is required for the amplification of SLCs/PLCs for LC pool. EGF exerts its action via binding to EGF receptor, because EGF antagonists (E and Cet) can completely reverse EGF‐mediated effects (Figure 1). Previous studies have demonstrated SLCs and PLCs as well as mouse LC cell lines (MA‐10) possessed both EGF receptors, and the LC was responsive to EGF stimulation.^13^, ^24^, ^26^, ^40^, ^44^, ^45^, ^46^ EGF stimulated the proliferation of SLCs/PLCs, possibly via the activation of Cyclin D1 (encoded by *Ccnd1*) and lymphoid enhancer factor (encoded by *Lef1*), as *Ccnd1* and *Lef1* were significantly up‐regulated in PLCs after EGF treatment. Cyclin D1 has been reported to be activated by EGF in various cells.^47^, ^48^ In the nucleus, LEF1 drives the expression of genes involved in cancer stem cell proliferation.^49^ LEF protein also mediates WNT signalling in adult tissues for stem cell proliferation.^49^ Interestingly, besides *Ccnd1*, other cell cycle regulatory genes (*Ercc1*, *Phlda11*, *Reln*, *Schip1* and *Cd82*) were also up‐regulated by EGF (Table 1). Apparently, SLCs did not possess LHCGR,^13^ and LH was not able to increase EdU incorporation into SLCs.^30^ Thus, at SLC stage, growth factors like EGF are critical for maintaining SLC pool via stimulating their proliferation.

Previous studies indicated that EGF activated phosphatidylinositol 3‐kinase (PI3K).^50^ PI3K is able to phosphorylate AKT1, thus in turn activating cyclin D1 pathway.^50^, ^51^ The data presented here show that EGF indeed increased AKT1 phosphorylation without affecting total AKT1 protein levels (Figure 7). This regulation of phosphorylation of AKT1 in PLCs is dependent on EGFR, because the EGF antagonist (E) can reverse EGF action (Figure 7).

The new data presented here also showed that activation of EGF inhibited H_2_O_2_‐induced apoptosis in PLCs (Figure 3). Indeed, EGF inhibited apoptosis in ILCs.^46^ The present data confirmed what was found for EGF action in ILCs. As AKT1 signalling mediates both proliferative and anti‐apoptotic effects, EGF‐mediated anti‐apoptotic action might be also mediated by AKT1.

Effects of EGF on T production in LCs are still controversial. Several in vitro studies found that EGF inhibited LH or cAMP‐induced steroid production in LCs.^52^, ^53^ Other studies indicated that EGF stimulated T production in LCs.^45^, ^46^, ^54^, ^55^, ^56^ The effects of EGF on androgen production depend on cell maturity, cell types of LCs, and EGF treatment duration.

In the current study, we clearly demonstrated that EGF blocked SLC/PLC differentiation into ALCs: (a) EGF concentration‐dependently lowered ALC number (Figure 2E) and medium T levels after induction of CD90^+^ SLC differentiating into ALCs in LDM (Figure 2) via EGFR signalling (Figure 2G and H); (b) EGF concentration‐dependently lowered AO and T production in PLCs (Figure 3A); (c) EGF down‐regulated *Hsd3b1* and *Star* expression in PLCs (Figure 5) and their protein levels (Figure 7A); and (d) EGF significantly down‐regulated *Pdgfra* (Figure 5), which was increased during Leydig cell development.^27^ EGF lowered the transcription of *Star*, which encodes StAR. StAR protein mediates the rate limiting in androgen synthesis, in which it serves the critical carrier of cholesterol to transport it for steroidogenesis from LC mitochondrial outer to the inner membrane.^57^, ^58^ After cholesterol is transported to inner membrane, CYP11A1 cleaves the cholesterol side‐chain to form pregnenolone. The exact mechanism of EGF‐induced down‐regulation of *Star* expression is unclear. Many signalling pathways, including protein kinase A, protein kinase C and nuclear receptor, are involved in the positive regulation of transcription of *Star* gene.^59^ EGF might interfere with one or more of these pathways. EGF also down‐regulated *Hsd3b1* (Figure 5)*,* which encodes HSD3B1, catalysing pregnenolone into progesterone. Interestingly, EGF‐mediated regulation of steroidogenesis in PLCs is quite different from LH, which actually up‐regulated all steroidogenesis‐related genes, including *Scarb1*, *Star*, *Cyp11a1*, *Cyp17a1* and *Hsd3b1*. LH primarily acts on LHCGR, thus activating cAMP/PKA signalling.

Interestingly, the expression levels of many genes in antioxidative proteins (*Ugt1a6*, *Idh1*, *Gsst1*, *Gstm2* and *Sod3*) were significantly down‐regulated more than twofold after EGF treatment. This could lead to ROS accumulation in PLCs (Figure 6). Indeed, EGF significantly led to ROS accumulation (Figure 3C). Several studies demonstrated that the accumulation of ROS is capable of damaging testis function^60^ and inhibiting T production by LCs.^61^ Mitochondrion is an important organelle for regulation of T synthesis and is sensitive to ROS attack.^62^, ^63^, ^64^ ROS is capable of disturbing StAR level.^65^ Although ROS accumulation might lead to cell apoptosis,^66^ the concentration of EGF (10 ng/mL) tested in the current study was not enough to induce PLC apoptosis (Figure 3). Actually, EGF can antagonize H_2_O_2_‐induced apoptosis (Figure 3).

TSP2 (encoded by *Tsp2*) was significantly down‐regulated by EGF (Figure 5). TSP2 functions the differentiation of mesenchymal stem cells into bones.^67^ The down‐regulation of *Tsp2* by EGF could lead to stem/progenitor Leydig cells stay in the Leydig cell lineage not into other cell lineage as SLCs were multipotent and SLCs can also differentiate into bone cells.^8^

In conclusion, the present study demonstrated that EGF induced the proliferation of SLCs/PLCs but blocks their differentiation into LCs.

## CONFLICT OF INTEREST

The authors report no conflicts of interest. The authors alone are responsible for the content and writing of the paper.

## AUTHOR CONTRIBUTIONS

Ren‐Shan Ge designed research; Xiaoheng Li, Yiyan Wang and Fei Ge performed research; Qiqi Zhu and Kaiming Yuan contributed new reagents; Ren‐Shan Ge and Zhijian Su analysed data, wrote the paper and revised the manuscript; and Yadong Huang supervised the experiments, revised and approved the manuscript.

## Supporting information

Table S1‐S2

## Data Availability

The data that support the findings of this study are available from the corresponding author upon reasonable request.
