# Supplementary material for: Epidermal growth factor regulates the development of stem and progenitor Leydig cells in rats
Source: J Cell Mol Med. 2020 May 22;24(13):7313–30. doi: 10.1111/jcmm.15302 (PMC7339176; doi:10.1111/jcmm.15302)
Supplement: Supplementary file 1 — Table S1‐S2 [file JCMM-24-7313-s001.doc]

**Supplementary Table S1**. Primer information

| **Primer**  **Symbol** | **Gene name** | **Primer direction** | **Sequences (5’to 3’)** | **PCR**  **(bp)** | **Accession** |
| --- | --- | --- | --- | --- | --- |
| Scarb1 | Scavenger receptor class B, member 1 | Forward | ATGGTACTGCCGGGCAGAT | 117 | NM_031541 |
|  |  | Reverse | CGAACACCCTTGATTCCTGGTA |  |  |
| Star | Steroidogenic acute regulatory protein | Forward | CCCAAATGTCAAGGAAATCA | 187 | NM_031558 |
|  |  | Reverse | AGGCATCTCCCCAAAGTG |  |  |
| Cyp11a1 | Cholesterol side chain cleavage enzyme | Forward | AAGTATCCGTGATGTGGG | 127 | NM_017286 |
|  |  | Reverse | TCATACAGTGTCGCCTTTTCT |  |  |
| Hsd3b1 | 3β-Hydroxysteroid dehydrogenase 1 | Forward | CCCTGCTCTACTGGCTTGC | 189 | NM_001007719 |
|  |  | Reverse | TCTGCTTGGCTTCCTCCC |  |  |
| Cyp17a1 | 17α-hydroxylase/ 17,20-lyase | Forward | TGGCTTTCCTGGTGCACAATC | 90 | NM_012753 |
|  |  | Reverse | TGAAAGTTGGTGTTCGGCTGAAG |  |  |
| Pdgfra | Platelet derived growth factor receptor alpha | Forward | ACCTTGCACAATAACGGGAG | 336 | M63837 |
|  |  | Reverse | CAGTTTGATGGACGGGAGTT |  |  |
| Gstm2 | Glutathione S-transferase mu 2 | Forward | GGATGCTCCCGACTATGACA | 113 | NM_177426 |
|  |  | Reverse | TGCTCTGGGTGATCTTGTGT |  |  |
| Ccnd1 | Cyclin D1 | Forward | cgcgtaccctgacaccaatct | 387 | NM_171992 |
|  |  | Reverse | cagaagcagttccatttgca |  |  |
| Mmp10  Thbs2  Lef1  Pcna  Rps16 | Matrix metallopeptidase 10  Thrombospondin 2  Lymphoid enhancer-binding factor 1  Proliferating Cell Nuclear Antigen  Ribosomal protein S16 | Forward  Reverse  Forward  Reverse  Forward  Reverse  Forward  Reverse  Forward  Reverse | ACCCCACTCACATTCTCCAG AAGCTCTGTCCCACTCCATC  GCTTCCACTGCCTGCCTTGTC  GCACGGATTCTCTGGCTCACATAC  CACACAACTGGCATCCCTCATCC  GGCTCCTGTTCCTTTCTCTGTTCG  AGGACGGGGTGAAGTTTTCT  CAGTGGAGTGGCTTTTGTGA  AAGTCTTCGGACGCAAGAAA  TTGCCCAGAAGCAGAACAG | 110  224  210  173  148 | NM_133514  NM_001169138  NM_130429  NM_022381  XM_341815 |

**Supplementary Table S2. Antibody information**

| **Antibody** | **Host species** | **Vendor (City, State)** | **Dilution** | |
| --- | --- | --- | --- | --- |
|  |  |  | **WB** | **IHC/IHF** |
| ACTB | rabbit | Cell Signaling Technology (Danvers, MA) | 1:1000 | ND |
| StAR | rabbit | Pterosaur Biotech (Hangzhou, China) | 1:1000 | ND |
| CYP11A1 | rabbit | Santa Cruz (Santa Cruz, CA) | 1:1000 | ND |
| HSD3B1 | rabbit | Abcam (San Francisco, CA) | 1:1000 | ND |
| AKT1 | rabbit | Abcam (San Francisco, CA) | 1:2000 | ND |
| pAKT1 | rabbit | Abcam (San Francisco, CA) | 1:5000 | ND |
| HSD11B1 | rabbit | Abcam (San Francisco, CA) | ND | 1:200 |
| SMA | mouse | Sigma Aldrich (Saint Louis, MO) | ND | 1:200 |

ACTB, β-actin; StAR, steroidogenic acute regulatory protein; CYP11A1, cytochrome P450 side chain cleavage; HSD3B1, 3-β-hydroxysteroid dehydrogenase 1; AKT1, serine/threonine kinase 1; pAKT1, phosphorylated serine/threonine kinase 1; HSD11B1, 11β-hydroxysteroid dehydrogenase 1; SMA, α-smooth muscle actin; ND = not detected.
